# Dentin Hypersensitivity: Etiology, Diagnosis and Treatment; A Literature Review

**Published:** 2013-09

**Authors:** AR Davari, E Ataei, H Assarzadeh

**Affiliations:** a Dept. of Operative Dentistry, School of Dentistry, Shahid Sadoughi University of Medical Sciences, Yazd, Iran.; b Postgraduate Student of Operative Dentistry, Dept. of Operative Dentistry, School of Dentistry, Shahid Sadoughi University of Medical Sciences, Yazd, Iran.

**Keywords:** Dentin Hypersensitivity, Etiology, Therapeutics, Diagnosis

## Abstract

The objective of this review is to inform practitioners about dentin hypersensitivity (DH); to provide a brief overview of the diagnosis, etiology and clinical management of dentin hypersensitivity and to discuss technical approaches to relieve sensitivity. This clinical information is described in the context of the underlying biology.

The author used PUBMED to find relevant English-language literature published in the period 1999 to 2010. The author used combinations of the search terms “dentin*”, “tooth”, “teeth”, “hypersensit*”, “desensitiz*”. Abstracts and also full text articles to identify studies describing etiology, prevalence, clinical features, controlled clinical trials of treatments and relevant laboratory research on mechanisms of action were used.

## Review

Many dentists have some problems in determining the etiology, diagnosing and treating dental hypersensitivity and some limitations have been observed in this regard. 

Some of the introducing titles relating to determination, diagnosis, prevalence, etiology and treatment of dental hypersensitivity are listed as follows:

*1- Definition*

Different terms have been used to describe dentin hypersensitivity a list of which has been shown in table 1 [1]. These terms are used based on the place of occurrence of hypersensitivity and include: cervical, root, dentine, cemental, and the terms sensitivity, and hypersensitivity [2-3]. All of these terms convey the same clinical conception and can be used interchangeably (Table 1). 

**Table 1:** Common terms which are used refer to dentin hypersensitivity

Dentin Hypersensitivity/ Sensitivity
Dentinal Hypersensitivity/ Sensitivity
Cervical Hypersensitivity/ Sensitivity
Root Hypersensitivity/ Sensitivity
Cemental Hypersensitivity/ Sensitivity

However, the term dentin hypersensitivity (DH) has been used in this article. According to the Canadian consensus document [4], DH has been defined as “pain derived from exposed dentin in response to chemical, thermal tactile or osmotic stimuli which cannot be explained as arising from any other dental defect or disease”. 

Although DH is a prevalent disorder and one of the most annoying diseases, the treatments which have been suggested for it are not sufficient and very successful [5]. This can lead to both physical and psychological problems for the patient. Furthermore, it can have a negative effect on the quality of a person’s life, especially with regards to dietary selection, maintaining optimal dental hygiene, and beauty aspects [6]. 

*2- Prevalence and epidemiology*

The prevalence distribution and appearance of the disease have been reported differently in different studies. These differences are due to the differences in populations, habits, dietaries, and methods of investigation [3]. 

Several studies have reported non-carious cervical lesions (NCCLs) and DH in adult populations, with prevalence rates ranging from 5% to 85% [7] and 2-8% to 74%, respectively [8]. 

The disease is prevalent in the patient with the age range of 20-50 years. However, it is more prevalent in the patient with the age range of 30-40 and more prevalent in female individuals that would probably be related to their dental hygiene and dietary [2-3, 5- 6, 9].

 There are two common methods to determine the intensity of DH. One of them is through asking some questions from the patient and the other is through clinical examination. The prevalence distribution of DH in the first method is usually estimated higher than that of the second method [10]. 

Furthermore, the occurrence of DH in canines and premolars is more than other teeth [2, 5-6, 8-9, 11]. The buccal surface of the teeth has been reported to be more involved with the disease than other places. Hence, dentists consider a prevalence distribution of 10-25% of DH for their patients and still consider it as a serious problem for 1% of them [12]. In some of the studies, it has been reported that DH can occur at any age [13]. 

It has been observed that some people with DH do not pursue treatment of the disease. However, they may report it in a clinical visit to the dentist. This is perhaps due to the fact that they do not consider DH as a (specific) disease [14]. 

*3- Etiopathogenesis*

3a- Anatomy of the tooth and dentin-pulp complex

Dentin is considered as a vital tissue and has the capacity to respond to physiologic and pathologic stimuli [15].

As it is known, dentin is covered by enamel in the crown surface and by a thin layer of cementum in the root surface of the tooth. Dentin is sensitive to stimuli due to the lesion extension of odontoblastic process and formation of dentin-pulp complex [1, 3, 6]. 

Dentin and pulp are histologically different. However, they have the same embryonic origin; ectomesenchymal origin. The formation of dentin-pulp causes dentin to be affected by pulp and vice versa. Dentin has very minute tubules which are filled with odontoblastic process. The processes are also surrounded by dentinal fluid which forms about 22% of the total volume of dentin. The fluid is completely filtrated and originates from the blood vessels of the pulp [13]. 

3b- Pathogenesis

Dentin’s sensitivity to stimuli does not lead to any problem while it is covered with protective tissues; enamel and cementum. The results of scanning electron microscope (SEM) indicate that the number of tubules in sensitive dentin is eight times more than the number of tubules in non sensitive dentin. Furthermore, tubules of sensitive dentin are thicker than those in non sensitive dentin [1, 3, 9, 13]. The rate of dentinal fluid flow depends on the fourth power of tubule’s radius and the difference is an important factor in the establishment of DH in the clinical conditions. 

Based on the studies, DH is developed in two phases [16-17]:

1. Lesion localization
2. Lesion initiation

In the first phase, dentinal tubules, due to loss of enamels, are exposed by attrition, abrasion, erosion, and abfraction. However, dentinal exposure mostly occurs due to gingival recession along with the loss of cementum on the root surface of canines and premolars in the buccal surface. It is worth noticing that not all the exposed dentins are sensitive. However, their calcified smear layer, as compared to non sensitive dentin, is thin and this leads to an increase in the fluid movement and consequently the pain response [9, 13, 18]. 

In the second phase, for the exposed dentin to be sensitized, the tubular plugs and the smear layer are removed and consequently, dentinal tubular and pulp are exposed to the external environment [17]. 

Plug and smear layer on the surface of exposed dentine are composed of elements of protein and sediments which are derived from salivary calcium phosphates and seal the dentinal tubules inconsistently and transiently. 

The findings of laboratory research indicate that both mechanical and chemical factors are effective in removing the smear layer from the dentinal tubules. However, the results of clinical investigations, the mechanical factors are not the only key factors in removal of the smear layer and when they are accompanied with acidic foods or drinks they lead to the removal of smear layer [3, 19]. 

It seems that Microbial plaque is not a significant factor in triggering DH [1]. First, as mentioned previously, the canines and first premolars have the greatest recession and sensitivity. The same teeth also reveal the lowest buccal plaque scores. Secondly, teeth with DH are cleaned extremely by patients suffering from the condition. This would suggest that plaque does not produce dentin hypersensitivity itself nor does it act as a stimulus for pain [20]. However, the effect of plaque on DH is a controversial issue [17].

4- Mechanism

**Figure 1:**
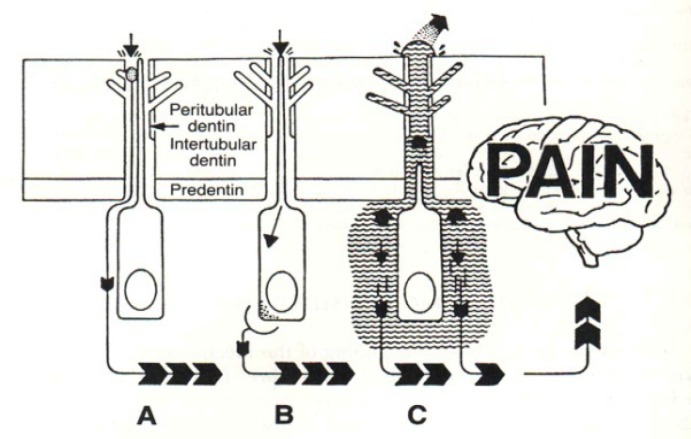
The schematic picture of the propped theories on DH

Three main mechanisms of dentin sensitivity are proposed [3]: (Figure 1).

- Direct Innervation (DI) Theory
- Odontoblast Receptor (OR) Theory
- Fluid Movement/Hydrodynamic Theory

Regarding the first theory; DI, it has been reported that the nerve’s endings enters dentin through pulp and extends to DEJ and the mechanical stimuli directly transmit the pain. However, there is little evidence to prove this theory; firstly because there is little evidence that can support the existence of nerve in the superficial dentin; where dentin has the most sensitivity; and secondly because the plexus of Rashkov do not become mature until complete tooth eruption. However, the newly developed teeth can be sensitive too [6].

In the OR theory, odontoblasts act as receptors of pain and transmit signals to the pulpal nerves. But this theory has also been rejected since the cellular matrix of odontoblasts is not capable of exciting and producing neural impulses. Furthermore, no synopsis has been found between odontoblasts and pulpal nerves [3]. 

Hydrodynamic Theory for sensitive dentine was first proposed by Brannstorm [21]. This theory is the most widely accepted theory for DH. The theory has been proposed based on the movement of the fluid inside the dentinal tubules. The theory claims that tubules are open between dentine surface which is exposed to the environment and pulp [21-22]. 

It is believed that DH is made as the result of movement of the fluid inside the dentinal tubules, which is further due to the thermal and physical changes, or as the result of formation of osmotic stimuli near the exposed dentine. The movement of fluid stimulates a baroreceptor and leads to neural discharge. The process is called the hydrodynamic theory of pain [3, 14]. This process is similar to activating the neural fibers around the hair by touching or pressing the hair. The movement of fluid can be toward the inside of the pulp or the outside of dentin. Cooling, drying, evaporation, and hypertonic chemical stimuli cause the dentinal fluid to flow away from the dentin-pulp complex and lead to an increase in pain [15]. 

Heating causes the fluid to flow toward the pulp. About 75% of patients with DH feel pain in response to cold stimuli [1-3]. 

As it was stated above, the number of tubules in sensitive dentin is eight times more than the number of tubules in non sensitive dentin. Furthermore, tubules of sensitive dentin are wider than those in non sensitive dentin.

*5- Clinical treatment of dentin hypersensitivity and Diagnosis*

As it is true about other diseases, the accurate diagnosis of dentin hypersensitivity before receiving treatment is critical for successful treatment. DH is similar to other conditions such as dentinal caries, fractured or chipped enamel, pain as a result of irreversible pulpitis, and post dental bleaching sensitivity in some of its features [1, 3, 23]. The diagnosis of the disease starts through investigating the medical history of the patient and examination. In investigating the medical history some questions are asked about the time of the start of DH, the intensity of the pain, the stability of the pain and the factors that reduce or increase the intensification of the disease.

In examination, some techniques such as pure air, pure water, and sounds are used in order to reconstruct the stimulating factors and to determine the degree of pain of the patient. Some other diagnostic tests are as follows: palpitation for diagnosing pulpitis or periodontal involvement, pushing a wood stick or transillumination for diagnosing a fracture or cracked tooth [24].

All of the teeth with pain should be examined and the degree of pain should be described through qualitative parameters such as slight, medium, and severe pain or through using quantitative parameters such as visual analogue scale [1, 3]. Eventually, all the characteristic data obtained from patient’s medical history and clinical examination can help to assess DH while ruling out all other causes of the pain. 

Removing etiologic factors and preventing DH

Before considering any treatment strategy for the management of DHS, it is important to note from the published literature that there are a number of individuals who may be at risk for dentin hypersensitivity [24] such as:

- Overenthusiastic brushers
- Periodontal treated patients

- Bulimics

- People with xerostomia

- High-acid food/drink consumers

- Older people exhibiting gingival recession

- Chewing ‘smokeless’ or ‘snuff’ tobacco

The often forgotten or neglected phase in the treatment of DH is the diagnosis and eliminating or treating the main routs of DH. The etiologic factors can be improper tooth brushing, premature occlusal contacts, gingival recession, and the existence of a large amount of exogenous and endogenous acids in diets. 

1. Improper tooth brushing; which includes using hard- or thick-bristle tooth brushes, brushing teeth with excessive pressure, excessive scrubbing at cervical areas or even missing to brush cervical areas [25]. To avoid the DH due to improper tooth brushing: The patient should be taught the correct method of tooth brushing [3].

- The patient should avoid the use of abrasive tooth pastes [1].
- The patient should avoid brushing at least for one hour after consuming acid drinks or foods (due to agonist effect of acidic erosion on tooth brush abrasion) [1, 3].

2. Premature contacts; sometimes through correction of occlusion or the use of an occlusal splint, the problem can be easily resolved [24].

3. Gingival recession; the patient should see a periodontist for consultation. Moreover, treatments such as graft or positioning flap might be adopted. 

4. Exogenous and endogenous acids (erosive agents)

It has been proved that erosive agents have a role in the initiation and progression of DH. These agents can open dentinal tubules through removal of the smear layer, tubules’ plugs and enamel [26]:

- Erosive agents with exogenous acids include carbonated drinks, citrus fruits, alcoholic drinks, yogurt, dairy products, and occupational hazard (such as workers in battery manufacturing plants) [3, 26].

The patient’s diet should be monitored for a while, concerning the quality and the frequency of consumption of acidic foods so that the necessary recommendations can be offered to the patient. Recommendations such as using of alkaline resources, like milk, or at least neutral materials, like water, after eating acidic foods, or having carbonated/acidic drinks with straw and avoiding to keep carbonated/acidic drinks in the mouth and tasting them [9].

- Erosive agents with endogenous acids enter the mouth through reflux or gastro-esophageal regurgitation [27]. These agents can be mostly found in patients with eating disorders. The patients are recommended to refer their doctors for the underlying diseases [3].

5. Poor oral hygiene contributes to periodontal diseases leading to root exposure. It has been also reported that periodontal treatment that exposes more root surface could increase incidence of DH [28].

**Classification of desensitizing agents [1, 3]**

1. Classification of desensitizing agents based on mode of administration
2. Classification of desensitizing agents based on mechanism of action

Classification of desensitizing agents which are used in treatment of DH is a challenging task. It is due to the fact that these agents are frequent. Furthermore, the mode of their action has not been determined yet. They can be easily classified into two groups based on the mode of their administration:

1. At home: this mode is simple and reasonable and can be used in treatment of many teeth.
2. In office: This is a complicated and expensive mode which can be used in treatment of a limited number of teeth.

Furthermore, the agents can be classified into two groups based on mechanism of action:

1. Those who disturb the neural response to pain stimulus
2. Those who block the flow of tubular liquid and therefore lead to occlusion of dentinal tubules.

Grossman [29] has itemized the characteristics of an ideal dentine desensitizing agent: rapidly acting, long-term effect, harmless to pulp, painless, easy to apply. Furthermore, such an agent does not stain the teeth. 

It is common that DH is treated based on occluding dentinal tubules or making sediments inside the tubules [1, 8]. 

At-home therapy

At- home desensitizing agents include tooth powders, tooth pastes, mouth washes and chewing gums.

1. Tooth dentifrice and tooth pastes: tooth pastes are amongst the most common *over-the-counter *(OTC) materials in desensitizing. When the desensitizing tooth pastes appeared on the market for the first time, they, those which contained strontium salt and fluoride, occluded dentinal tubules. However, those which contained formaldehyde, destroy vital elements inside the tubules. 

Nowadays, most of the desensitizing toothpastes contain potassium salts such as potassium chloride, potassium citrate, and potassium nitrate [9]. The studies have revealed that potassium salts move along the dentinal tubules and through blocking the axonic action of the intra-dental nerve fibers decrease the excitability of the tooth.[3, 30].

Different studies have been performed on potassium salts and their effect on DH. Based on these studies, the use of tooth pastes which contain potassium nitrate and fluoride has a positive effect on reducing DH [1, 3, 5-6, 13]. These tooth pastes should be used with soft-bristled tooth brushes. Furthermore, the patients are recommended to use the minimum amount of water so that the tooth pastes would have their maximum positive effects [3]. 

Several studies have also shown that remineralizing tooth pastes which contained sodium fluoride and calcium phosphates could reduce DH dramatically [31-33]. 

Recently, some tooth pastes and powders contain arginine and are proved to be effective through several clinical studies. They contain 8% arginine, calcium carbonate, and 1450 ppm fluoride and by establishing an alkaline environment, lead to the precipitation of more salivary calcium and phosphate on the surface and within the dentinal tubules. Furthermore, calcium carbonate, through attracting arginine, forms a molecule which is positively charged [11]. 

Toothpastes with different ingredients and different concentrations of desensitizing agents and other agents such as anti-plaques and abrasives may have opposite effects on DH. However, in two of the studies which were done in 2005, these agents did not have a significant effect on the desensitizing property of the tooth pastes under study [1]. 

The dentist should teach the patient the correct method of tooth brushing.

Tooth powders should also be used with soft-bristled tooth brushes. There is no evidence to indicate a better result in using these powders through using fingers instead of tooth brushes [1]. 

2. Mouthwashes and chewing gums:

Findings indicate that mouthwashes which contain potassium nitrate and fluoride reduce DH. 

A few studies have also been done on the chewing gums containing potassium chloride. However, the results of such studies are not much reliable [1, 14]. 

About 2-4 weeks after at- home therapies, the degree of DH would be reinvestigated. If the pain still existed, the patient should start the next phase of the therapy; in-office therapy [1-3]. 

In-office therapy

Theoretically, in-office therapy of DH should lead to immediate relief of the pain. However, practically, this might not be the case [3]. Classification of different types of clinical desensitizing agents is based on their mechanism of action and includes occluding dentinal tubules and disturbing the transmission of nerve impulses.

Disturbing the transmission of nerve impulses agent

Only potassium salts (potassium nitrate) and perhaps laser can be placed into the group of disturbers of nerve impulse transmission. Before the discovery of local anesthesia, the dentists used a series of chemical materials such as silver nitrate, zinc chloride, and arsenic compounds to alleviate dentine discomfort. Nowadays, less poisonous materials are used for desensitizing [1, 17].

Potassium nitrate

It is available in two forms of aqueous solution and adhesive gel. As it was stated above, the number of potassium ions decrease when they enter dentinal tubules and decrease the excitability of nerves that transmit pain [3, 

6, 13]. 

Occluding dentinal tubules agents

Fluorides

There are many articles on the effectiveness of fluorides in decreasing DH. Fluorides precipitate calcium fluoride crystals inside dentinal tubules, and thus decrease dentinal permeability [16]. These crystals are almost insoluble.

Sodium fluoride with a 2 % concentration is used in the office. The precipitate which is formed by sodium fluoride can be removed by the saliva or mechanical scrubbing. Therefore, acid has been added to the formula so that the resultant acidulated sodium fluoride can form precipitates deep in the tubules [3]. 

Fluorides and fluoro-silicates can be used in combination with iontophoresis, which through electrical current can increase ionic diffusion [2]. 

Stannous fluoride has the same effect as sodium fluoride. If the precipitate of apatite fluoride forms, it can resist against salivary action, tooth brushing and dietary substances’ action [34-35]. 

Oxalates

Oxalates can occlude dentinal tubules and reduce permeability of dentine, up to 98% [36-37]. The application of 28% potassium oxalate can lead to the formation of calcium oxalate in the depth of dentinal tubules. However, findings have indicated that the reduction of dentin hypersensitivity induced by oxalate, remains for a short time. To increase the effectiveness of oxalate, the surface of the tooth can be etched [38]. Potassium oxalate can lead to some digestive disorders so it should not be used for a long term [3]. 

Varnishes

Varnishes can act as a means to help other materials increase their therapeutic effect. Fluoride varnishes combine with acid to increase its effectiveness [39-40]. 

Copal varnish is used to cover the exposed dentine. However, its effect remains for a short period of time and it needs to be applied several times [40]. 

Adhesive resins

Adhesive systems, unlike the other local desensitizing agents which have a short-term effect, exhibit a long-term or permanent effect. These adhesives include varnishes, bonding agents, and repairing resin composites. The composites can effectively seal dentinal tubules through forming a hybrid layer [1]. 

The old adhesives formed the hybrid layer through removing the smear layer and etching the dentinal sur-

face so that deep resin tags could be formed [41-42]. The new adhesives, however, act in a way that the smear layer will be modified and incorporated into the hybrid layer [43]. It is claimed that the recent dentin bonding agents (DBA) can manage or prevent DH. For example, Gluma desensitizing agent (Heraeus Kulzer; Ca, USA) includes hydroxyethyl methacrylate (HEMA), benzalkonium chloride, gluteraldehyde and fluoride. Gluteraldehyde can lead to protein coagulation within dentinal tubules. HEMA can cause resin tags to be formed and dentinal tubules to be occluded [3, 43]. Gluma has shown good results in DH management in clinical trials [43]. 

Bioglass

Bioglass has been produced to stimulate bone formation. It is employed to fill the osseous defects during periodontal surgery [44]. There are some reports which indicate the effectiveness of bioglass in mineralization and infiltration of dentinal tubules. Its main component is silicate which acts as a nucleus for precipitation of calcium and phosphate. Scanning electron microscopic (SEM) analysis has shown that the application of bioglass causes the formation of an apatite layer which further leads to the occlusion of dentinal tubules [45-46]. 

Portland cement 

Some researchers have shown that silicate cement which is derived from Portland cement can be effective in DH management and help the occlusion of tubules through Remineralization [2]. 

Casein-phosphopeptide-amorphous calcium phosphate (CPP)-(ACP)

Recently, a remineralizing agent has been produced out of milk casein proteins and has appeared on the market under the name GC Tooth Mousse (GC Asia Pty. Ltd.; Japan). CPP containing phosphoseryl sequences can be helpful in attaching and stabilization of ACP. CPP-ACP remineralizes the early lesions of enamel subsurface [47]. The manufacturing factory has claimed that the product can be effective in prevention and treatment of DH. 

Laser

According to previous researches, the effect of laser on the treatment of DH is different and might be between 5-100% based on the type of laser and therapeutic parameters such as the laser’s length of beam; the amount of time spent on the use of laser; and the intensity of laser [48-50]. Different mechanisms of action have been proposed for laser, its effect on the dentine and its effect on reducing DH. They include [50]: 

1. Occlusion through coagulation of the proteins of the fluid inside the dentinal tubules 

2. Occlusion of tubules through partial sub-melting

3. Discharging of internal tubular nerve 

In a systematic review of the articles, published through the years 2000-2010, on the effects of laser therapy on treating DH, it has generally been claimed that laser therapy for the treatment of DH is preferred to other relevant local therapies [50]. However, further clinical long-term studies in many different samples and better qualities need to be done to prove this claim [49]. In addition, this type of therapy is highly acceptable to patients because its proper usage has no negative impacts. So far, there has been no report of adverse reactions or pulp damage in the studies. Thus the use of laser in treatment of DH is both logical and acceptable [49-50]. 

The patient must be informed in different phases of the therapy:

1. Taking a detailed clinical and dietary history

2. Correct diagnosis of the condition with concerning the differential diagnosis of other painful conditions. Diagnosing and treating of the probable etiologic factors causing DH

3. Initiating the at-home treatment in cases of mild-to-moderate sensitivity 

4. Initiating in-office treatment in cases of severe sensitivity or when one or two teeth were involved

5. Considering RCT in cases were at-home and in-office treatments were not effective or when a number of teeth display the symptoms of DH

5. Organizing regular follow-up visits with an emphasis on the prevention of DH 

## Discussion

Dentin hypersensitivity as a chronic disease is increasingly prevalent among adults and some researches has been done on determining etiologic factors in causation of the disease, its diagnosis and its treatment [1, 6]. This disorder usually occurs as the result of loss of enamel and cementum or exposure of dentinal tubules [1, 3-4, 9]. The intensity and degree of sensitivity depends on different factors and are different in different people [6]. There have been proposed many materials and methods in order to reduce or remove sensitivity. They include the use of tooth pastes containing potassium salts, fluoride composites, resins, laser; bioglass and so on. These materials usually exert their effects through sealing dentinal tubules or through disturbing the transmission of nerve impulses [1-2, 6, 24]. 

Etiologic factors have been underestimated by dentists or specialists in treatment of DH.. [1]. 

Based on the performed studies, different theories have been proposed on the dentin hypersensitivity of which direct innervation theory and odontoblast receptor theory have faced some challenges. However, Brannstorm theory which deals with the flow of fluid inside the dentinal tubules is more realistic [3, 21]. 

Still, the most common therapy and usually the first therapy in treating dentin hypersensitivity is the use of tooth pastes containing potassium salts and fluoride [1, 6].The new offered materials and methods, such as bioglasses, CPP-ACP, laser, iontophoresis, and even homeopathy for the treatment of DH have been tested through different studies and the obtained findings have been different [5-6, 9-12, 30]. 

The use of laser for the treatment of dentin hypersensitivity is highly acceptable among patients. Furthermore, the use of it has no later negative impact and this is promising [49-50]. 

*Treatment strategy of DH for dentists; based on the patient complications*

1. Diagnosing and treating of the probable etiologic factors in causation of DH
2. In cases of mild-to-moderate sensitivity, after teaching the patient the correct method of tooth brushing, initiate the at-home treatment for example: prescribe desensitizing tooth pastes containing calcium, potassium, fluoride (at least 1450 PPM) salts and argenin with using soft-bristled tooth brushes or prescribe fluoride mouth washes,
3. If the pain was still felt, about 2-4 weeks after at home therapies, in cases of severe sensitivity or when one or two teeth are involved; initiate in-office treatment in sequence, include: application of acidulated phosphate fluoride (APF) gel, fluoride varnish, Gluma desensitizing agent, GC Tooth Mousse (CCP-ACP) and laser therapy.
4. Finally, Considering RCT in cases where at-home and in office treatments were not effective.
5. Organizing regular follow-up visits with an emphasis on the prevention of DH.

## Conclusion

The present study is a non-systematic review of the literature concerning the etiology, diagnosis and management strategies of DH. 

The aim of this study was to increase the knowledge of dental trainees, dentists and specialists in the field of dentistry regarding the dentin hypersensitivity. The current review has presented the newest trend on the etiology, methods of diagnosis, and management strategies of the disease. It should, however, be mentioned that the future research on DH may entail some revisions, modifications or changes in the content of the current study. 

DH can be detected after removing all the other factors which can possibly cause the pain? The treatment of dental hypersensitivity should be on a regular basis and initiate with at-home therapy and then continue with complementary therapies. It is recommended that follow-up visits should be organized for all the patients after undergoing periodic treatments. 
